# ASSOCIATIONS BETWEEN HEARING LOSS AND HEALTH CARE UTILIZATION: EVIDENCE FROM NHATS

**DOI:** 10.1093/geroni/igad104.0414

**Published:** 2023-12-21

**Authors:** Emmanuel Garcia, Nicholas Reed

**Affiliations:** Johns Hopkins Bloomberg School of Public Health, Baltimore, Maryland, United States; Johns Hopkins Bloomberg School of Public Health, Baltimore, Maryland, United States

## Abstract

We investigated the association between self-reported hearing loss and healthcare utilization among community dwelling older Medicare beneficiaries (age ≥65) who were enrolled in the 2011 cycle of the National Health and Aging Trends Study (NHATS). Participants’ survey data were linked to fee-for-service Medicare claims data during the 2011-2016 period. Hearing loss was ascertained using based on participants’ hearing aid use, and difficulty hearing in various noisy situations. Those who reported any difficulty hearing or reported hearing aid use were categorized as having hearing loss. Healthcare utilization included: (1) number of inpatient hospitalizations, (2) number of Emergency Department visits, and (3) total number of days spent during hospitalizations per calendar year. Each participant with hearing loss was matched to a participant without hearing loss using a one-to-one propensity score matching to minimize confounding bias. Using generalized estimating equations, we found that among our matched sample those with hearing loss had 21% (Incidence Rate [IR]=1.21; 95% Confidence Interval [CI]= 1.06,1.37) higher rates of emergency department visits, and 48% (IR = 1.48; 95% CI: 1.20,1.82) higher number of days spent in a hospital over the 6-year period from 2011-2016. No association was found between hearing loss and the number of hospitalizations. These finding suggest that older adults with hearing loss may be at risk for increased healthcare utilization. Interventions for hearing loss, such as the use of hearing aids, might reduce healthcare utilization and associated expenditures among older adults in the U.S. Medicare program.

